# Inhibitory Effects of Sesquiterpenoids Isolated from *Artemisia scoparia* on Adipogenic Differentiation of 3T3-L1 Preadipocytes

**DOI:** 10.3390/ijms25010200

**Published:** 2023-12-22

**Authors:** Jung Im Lee, Jung Hwan Oh, Fatih Karadeniz, Chang-Suk Kong, Youngwan Seo

**Affiliations:** 1 Incheon Regional Office, National Fishery Products Quality Management Service Incheon, Incheon 22346, Republic of Korea; jimlee@korea.kr; 2 Nutritional Education, Graduate School of Education, Silla University, Busan 46958, Republic of Korea; jhoh@silla.ac.kr; 3 Marine Biotechnology Center for Pharmaceuticals and Foods, Silla University, Busan 46958, Republic of Korea; karadenizf@silla.ac.kr (F.K.); cskong@silla.ac.kr (C.-S.K.); 4 Department of Food and Nutrition, Silla University, Busan 46958, Republic of Korea; 5 Division of Convergence on Marine Science, Korea Maritime and Ocean University, Busan 49112, Republic of Korea

**Keywords:** 3T3-L1, adipogenesis, *Artemisia scoparia*, reynosin, santamarine

## Abstract

Obesity and related complications are significant health issues in modern society, largely attributed to a sedentary lifestyle and a carbohydrate-rich diet. Since anti-obesity drugs often come with severe side effects, preventative measures are being sought globally, including dietary changes and functional foods that can counteract weight gain. In this context, plant-based metabolites are extensively studied for their advantageous biological effects against obesity. Several plants within the *Artemisia* genus have been reported to possess anti-adipogenic properties, preventing adipocytes from maturing and accumulating lipids. The present study investigated the anti-adipogenic potential of two sesquiterpenoids, reynosin and santamarine, isolated from *A. scoparia* in adipose-induced 3T3-L1 preadipocytes. Differentiating 3T3-L1 adipocytes treated with these isolated compounds displayed fewer adipogenic characteristics compared to untreated mature adipocytes. The results indicated that cells treated with reynosin and santamarine accumulated 55.0% and 52.5% fewer intracellular lipids compared to untreated control adipocytes, respectively. Additionally, the mRNA expression of the key adipogenic marker, transcription factor PPARγ, was suppressed by 87.2% and 91.7% following 60 μM reynosin and santamarine treatment, respectively, in differentiated adipocytes. Protein expression was also suppressed in a similar manner, at 92.7% and 82.5% by 60 μM reynosin and santamarine treatment, respectively. Likewise, SERBP1c and C/EBPα were also downregulated at both gene and protein levels in adipocytes treated with samples during differentiation. Further analysis suggested that the anti-adipogenic effect of the compounds might be a result of AMPK activation and the subsequent suppression of MAPK phosphorylation. Overall, the present study suggested that sesquiterpenoids, reynosin, and santamarine were two potential bioactive compounds with anti-adipogenic properties. Further research is needed to explore other bioactive agents within *A. scoparia* and elucidate the in vivo action mechanisms of reynosin and santamarine.

## 1. Introduction

Due to Westernized dietary patterns and changes in lifestyle, obesity rates have been continuously rising worldwide, and the incidence of obesity-related diseases has also increased accordingly [1,2]. Obesity is a metabolic disease caused by an imbalance between energy accumulation and consumption in the body and appears as homeostasis in the body is disrupted by mental, physical, and environmental factors. Obesity has been known to be a cause of various metabolic syndromes, cardiovascular diseases, and diabetes, and various anti-obesity drugs have been developed worldwide to prevent and treat it [3]. However, drugs for treating obesity were reported to be accompanied by serious side effects due to excessive nervous system stimulation. Recently, active research has been focused on developing nutraceuticals for effective weight control and revealing their mechanism of action [4,5] using in vitro models, such as preadipocytes. When the preadipocytes were treated with dexamethasone and 3-isobutyl-1-methylxanthine, the expression of CCAT/enhancer-binding protein beta (C/EBPβ), an early differentiation transcription factor, was stimulated. Then, the expressed C/EBPβ induced lipogenesis by sequentially increasing the expression of peroxisome proliferator-activator gamma (PPARγ) [6,7]. In the middle and late stages of adipogenesis, fat biosynthesis took place due to the activation of the transcription factor sterol regulatory element-binding protein 1c (SREBP1c), and lipogenic enzymes increased to accumulate lipid droplets of intracellular triglycerides, thereby completing adipocyte differentiation [8]. Studies showed that hindering this process resulted in fewer adipocytes and, therefore, smaller adipose tissue [9,10,11].

Salt plants are the plants that live in a salt marsh, a unique intermediate zone where water and land meet and where the influx of organic and inorganic salts is mixed. Salt plants have been known to have unique metabolic processes to protect cells from damage caused by the accumulation of reactive oxygen species, which were generated in vivo due to the high salinity of salt marshes. Therefore, to maintain ion homeostasis, these plants have been producing various secondary metabolites that are distinct from those of terrestrial plants, and a variety of bioactivities such as anti-cancer, anti-aging, anti-inflammation, and antioxidant have been reported for these metabolites [12,13,14]. Among them, species belonging to *Salicornia* and *Artemisia* have been studied for their anti-adipogenic effects in both in vitro and in vivo models. *Salicornia arabica* [15], *Salicornia europaea* [16], and *Salicornia herbacea* [17] were all shown to express anti-adipogenic effects by inhibiting PPARγ-mediated adipogenic differentiation and lipid accumulation. In addition, *Artemisia annua* [18], *Artemisia campestris* [19], and *Artemisia princeps* [20] were all suggested to possess anti-obesity effects by inhibiting adipogenesis. Apart from these species, several other halophytes, such as *Nitraria retusa* [21], *Adenocaulon himalaicum* [22], and *Limonium tetragonum* [23,24], also showed anti-obesity potential, specifically by acting on the differentiation of adipocytes.

*Artemisia scoparia*, a perennial plant of the Asteraceae family, is a halophyte that grows in small groups in coastal tidal flats or sandy salt marshes, as well as in dry areas [25]. It is distributed in Korea, Japan, Mongolia, Russia, Northern India, Central Asia, and Europe. Young shoots of *A. scoparia* have been traditionally used in folk medicine to treat fever, gout, jaundice, and urinary and skin disorders [25,26]. Flavonoids, coumarins, phenols, alkynes, terpenoids, sterols, and essential oils [27,28] have been isolated from *A. scoparia*, and various biological activity studies, such as anti-cancer, antiviral, anti-inflammatory, anti-diabetic, and antibacterial, have been published testing *A. scoparia* extracts and active compounds [28]. However, a study reported that in the absence of any adipogenic trigger, *A. scoparia* extract enhanced adipogenesis in vitro to exert an anti-diabetic effect [29] in addition to adipogenic activator compounds, such as prenylated coumaric acids [30]. Although *A. annua* has been widely studied for its chemical composition and anti-obesity effect [18], and the literature has been lacking a similar effort on *A. scoparia*. Therefore, in this study, we investigated the inhibitory effect of the two sesquiterpenoid constituents from *A. scoparia* on adipogenesis and the signaling mechanism underlying this effect.

## 2. Results

### 2.1. Isolation and Identification of the Compounds

**Figure 1:**
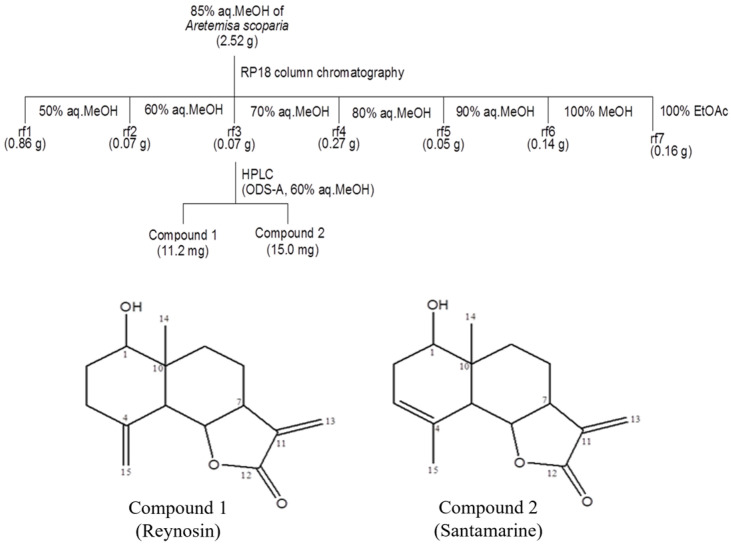
Isolation scheme and chemical structures of compounds reynosin and santamarine from the halophyte *Artemisia scoparia*.

The 85% aqueous methanol (aq. MeOH) fraction of the extract from *A. scoparia* was processed through vacuum flash chromatography on C_18_ silica gel. Seven subfractions were obtained by employing a solvent gradient of MeOH and water as eluents. The reversed-phase high-performance liquid chromatography (HPLC) separation of the third subfraction (Fr. 3), obtained in 70% aq. MeOH, resulted in the isolation of two compounds, reynosin and santamarine (Figure 1). The chemical structures of the isolated compounds were identified by comparing nuclear magnetic resonance (NMR) spectroscopic data with those reported in the literature [31].

### 2.2. Cytotoxicity of the Compounds

**Figure 2:**
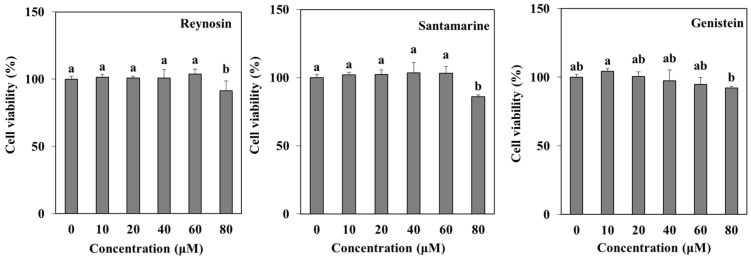
The cytotoxic effect of reynosin, and santamarine from *A. scoparia* on 3T3-L1 preadipocytes. 3T3-L1 preadipocytes were treated with 10, 20, 40, 60, and 80 μM of the three samples for 24 h. Cell viability was determined using MTT analysis. ^a–b^ Identical letters indicate an absence of a statistically significant difference (*p* > 0.05).

Cells were treated with 10, 20, 40, 60, and 80 µM of reynosin and santamarine, respectively, along with genistein used as a positive control to inhibit adipogenesis. The effect of increasing concentrations of samples on cell survival was measured. As a result, in the case of reynosin and genistein, the cell viability rate was over 90% at concentrations of 10, 20, 40, 60, and 80 μM. Santamarine showed more than 100% cell viability at all concentrations except 80 μM (Figure 2). The LC_50_ values for reynosin and santamarine were calculated to be 220.4 μM and 188.6 μM, respectively. Based on the above results, the inhibitory effect of these compounds on adipogenesis was confirmed using samples up to a concentration of 60 μM, which did not significantly affect cell viability.

### 2.3. Inhibition of Lipid Accumulation by Reynosin and Santamarine

**Figure 3:**
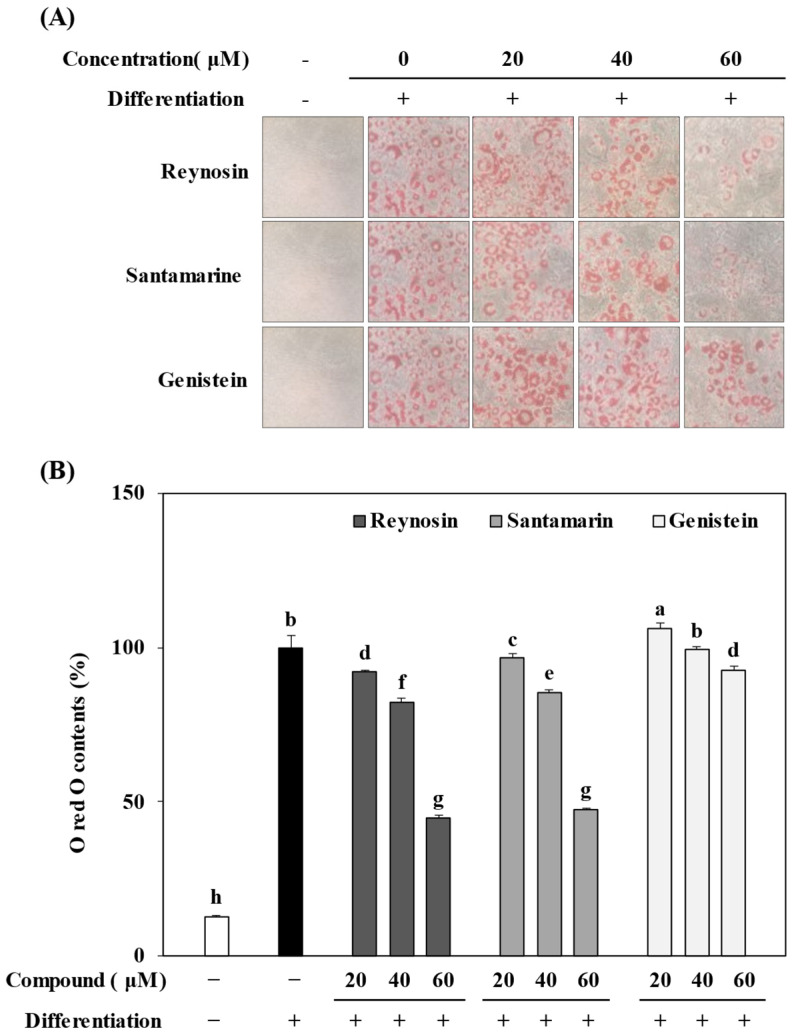
Effect of reynosin and santamarine on the lipid accumulation of differentiated 3T3-L1 adipocytes. Differentiation of confluent 3T3-L1 mouse preadipocytes was initiated with a differentiation-inducing medium containing a normal feeding medium supplemented with 0.5 mM 3-isobutyl-1-methylxanthine, 0.25 μM dexamethasone, and 5 μg/mL insulin for 2 days. The adipogenic characters were maintained by a differentiation medium (a feeding medium supplemented with 10% FBS, 5 μg/mL insulin) in the absence or presence of samples for 8 days. (**A**) The non-differentiated and differentiated adipocytes were fixed and stained with Oil Red O to visualize lipid droplets by light microscopy (magnification ×200). (**B**) Stained oil droplets were eluted from cells with isopropanol and quantified by spectrophotometric analysis at 500 nm. ^a–h^ For each group of values, different letter denotations indicate statistically significant differences, whereas the same letters indicate otherwise (*p* > 0.05).

In order to investigate the effects of reynosin and santamarine on the accumulation of intracellular lipid droplets during the adipogenic differentiation of 3T3-L1 preadipocytes, the preadipocytes were treated with concentrations of 20, 40, and 60 μM of each compound separately. Subsequently, the degree of triglyceride accumulation in adipocytes was evaluated using Oil Red O staining (Figure 3). In the control group, where adipogenesis was induced without sample treatment, a marked increase in the formation of intracellular triglycerides was observed. However, upon treatment with reynosin and santamarine at a concentration of 60 μM each, intracellular triglyceride levels decreased by 55.0% and 52.4%, respectively, compared to the control group. These results indicated a higher inhibitory efficacy than genistein, a positive control group, demonstrating significant inhibition of lipid accumulation by reynosin and santamarine.

### 2.4. Inhibition of mRNA Expression of Adipogenic Markers by Reynosin and Santamarine

**Figure 4:**
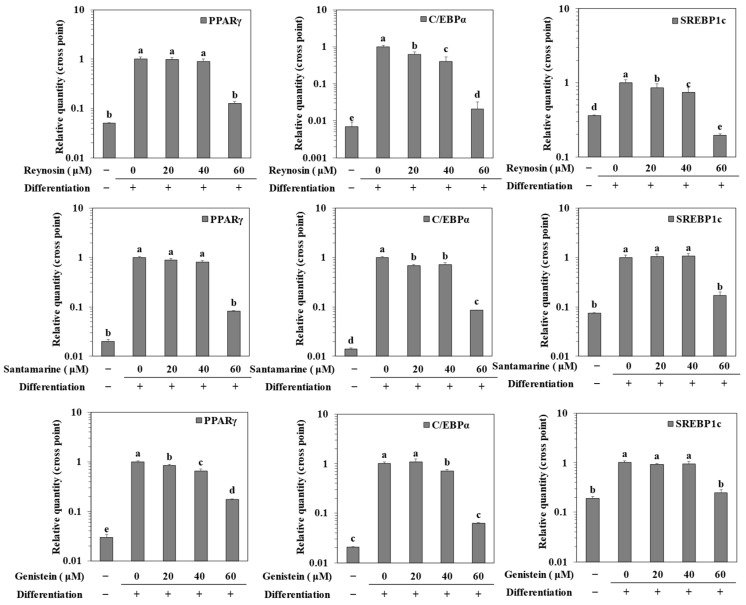
Effect of reynosin and santamarine on the mRNA expression levels of key adipogenic differentiation markers, PPARγ, C/EBPα, and SREBP1c, in 3T3-L1 adipocytes. Differentiation of confluent 3T3-L1 mouse preadipocytes was induced with the absence or presence of samples for 8 days. Total cellular RNA was extracted, and reverse transcription was performed. Synthesized cDNA was subjected to quantitative PCR. ^a–e^ For each group of values, different letter denotations indicate statistically significant differences, whereas the same letters indicate otherwise (*p* > 0.05).

The effect of reynosin and santamarine on the expression of adipogenic transcription factors was confirmed at the mRNA level through a semi-quantitative reverse transcription polymerase chain reaction (RT-qPCR). Compared to preadipocytes without differentiation, the expression of representative adipogenesis-related genes (C/EBPα, PPARγ, and SREBP1c) significantly increased in the differentiated but non-treated control group. However, treatment with reynosin and santamarine significantly reduced the mRNA expressions of the aforementioned genes (Figure 4), similar to the effect of the positive control genistein. Although the degree of the downregulation of transcription factors varied, the anti-obesity effect was clearly established.

### 2.5. Inhibition of the Expression of Adipogenic Marker Proteins by Reynosin and Santamarine

**Figure 5:**
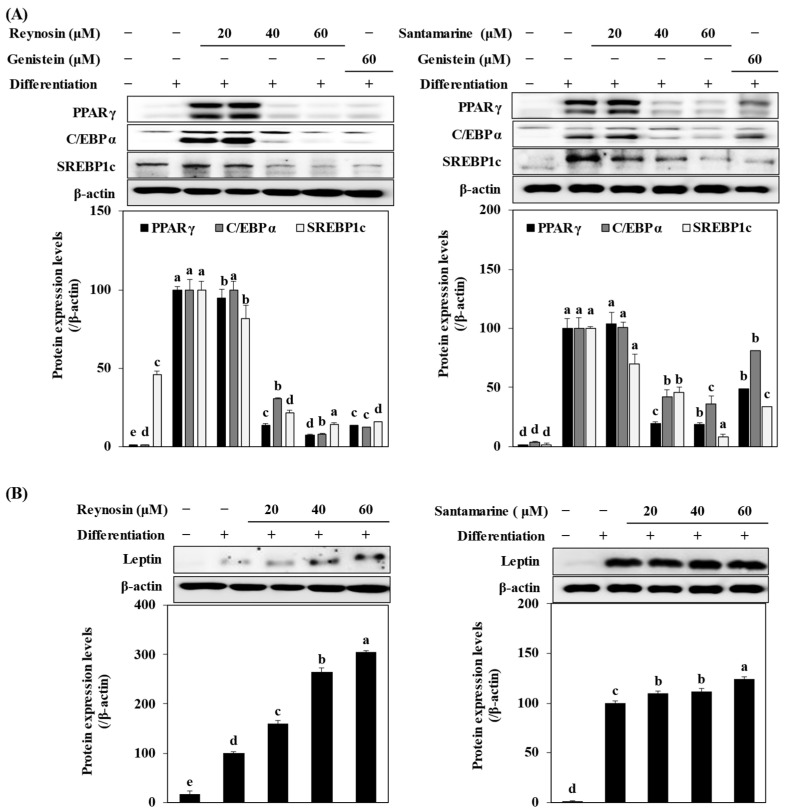
Effect of reynosin and santamarine on the protein expression levels of (**A**) adipogenic transcription factors and (**B**) adipocyte-specific protein and leptin in differentiated 3T3-L1 adipocytes. Differentiation of confluent 3T3-L1 mouse preadipocytes was induced with the absence or presence of samples for 8 days. On day 8, the cells were lysed, and cellular proteins were separated by SDS-polyacrylamide gels and transferred onto nitrocellulose membranes. The membranes were examined with the indicated antibodies. Protein bands were visualized using an ECL detection system. β-actin served as an internal control. ^a–e^ For each group of values, different letter denotations indicate statistically significant differences, whereas the same letters indicate otherwise (*p* > 0.05).

The effect of reynosin and santamarine on the expression of adipogenic transcription factors and adipogenesis-related genes involved in adipocyte maturation was confirmed at the protein level through Western blot. First, when examining the expression levels of adipogenic transcription factors, it was observed that the expression of C/EBPα, PPARγ, and SREBP1c was significantly elevated in the untreated differentiated control group compared to preadipocytes without induced differentiation. However, treatment with reynosin and santamarine led to a substantial reduction in their expressions (Figure 5A). Furthermore, the levels of leptin, an adipocyte-specific protein, also significantly decreased following treatment with reynosin and santamarine (Figure 5B). Based on these findings, it could be inferred that reynosin and santamarine suppressed the levels of adipogenic transcription factors, consequently leading to a decreased production of leptin, which was associated with the accumulation of lipid droplets and gained adipocyte characteristics.

### 2.6. Inhibition of MAPK Signaling by Reynosin and Santmarine

**Figure 6:**
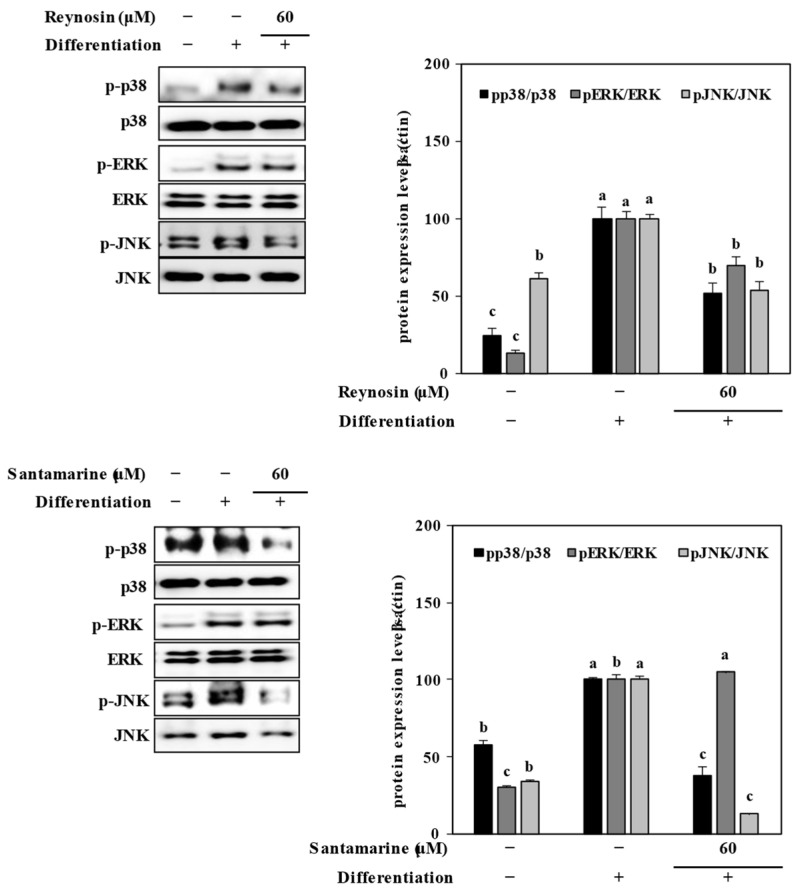
Effect of reynosin and santamarine on the total and phosphorylated (p-) protein levels of the MAPK pathway in 3T3-L1 adipocytes. The data represent the mean ± SD of three separate experiments. ^a–c^ For each group of values, different letter denotations indicate statistically significant differences, whereas the same letters indicate otherwise (*p* > 0.05).

To elucidate the mechanisms underlying the inhibition of adipogenesis by reynosin and santamarine, the signaling pathway of mitogen-activated protein kinase (MAPK) was investigated. After 8 days of differentiation, the protein expression levels of total and phosphorylated p38, extracellular signal-regulated kinase (ERK), and c-Jun N-terminal kinase (JNK) were confirmed in adipocytes using Western blot. In the untreated differentiated control group, the phosphorylation of all MAPK members increased, but when treated with reynosin, the phosphorylation levels were suppressed in the differentiated 3T3-L1 preadipocytes (Figure 6). Additionally, treatment with santamarine led to a decrease only in the phosphorylation levels of p38 and JNK.

## 3. Discussion

When differentiation is induced in 3T3-L1 preadipocytes, intracellular triglycerides are accumulated as lipid droplets along with the maturation of the adipocytes [32]. Adipogenesis, the process that transforms preadipocytes into adipocytes, is quite complex and involves changes in cell morphology, gene expression, and hormone sensitivity. Multiple types of adipogenic transcription factors are known to participate in this process. During the initial stages of differentiation, the expression of C/EBPβ and SREBP1 is induced by differentiation-inducing factors. C/EBPβ promotes the expression of C/EBPα and PPARγ, which contribute to the development of mature adipocytes with characteristics, such as insulin-sensitive glucose uptake and the accumulation of intracellular lipid droplets. Similarly, SREBP1c, activated by insulin, directly influences the expression of PPARγ [33,34]. The MAPK signaling pathways encompass p38, ERK1/2, and JNK1/2, all of which are recognized to play roles in adipocyte differentiation and proliferation [35,36,37]. Activated p38, ERK1/2, and JNK1/2 proteins contribute to the activation of PPARγ expression, leading to the formation of a heterodimer with a retinoic acid-like receptor. This complex then binds to peroxisome proliferator response elements in target genes, ultimately inducing adipogenesis. Based on the findings mentioned above, it has been established that reynosin and santamarine hinder adipogenesis by reducing the expression of adipogenic transcription factors through the inhibition of MAPK signaling, achieved by suppressing phosphorylation. The preadipocytes treated with both compounds expressed significantly reduced levels of PPARγ, C/EBPα, and SREBP1c in both mRNA and protein levels. The effect was similar to Genistein, the positive control, which was reported to inhibit adipogenesis [38,39]. This suppression was evidenced by the lack of adipocyte characteristics in cells treated with compounds that inhibited lipid accumulation and leptin expression. Compared to the untreated differentiated control group where MAPK activation was stimulated during differentiation, cells treated with reynosin and santamarine exerted lower phosphorylation of p38 and JNK MAPKs. These results highlighted the potential of reynosin and santamarine, two sesquiterpenoids from *A. scoparia*, for controlling obesity and related metabolic disorders by preventing adipose tissue formation. Moreover, the outcomes of this study were anticipated to hold significant value as valuable data for ongoing research focused on the exploration of functional substances derived from halophytes in the future.

Sesquiterpenoids, a sub-family of sesquiterpenes to which reynosin and santamarine belong, are highly bioactive secondary metabolites. Studies reported several bioactivities for sesquiterpenoids on a wide range of in vitro models. Oh et al. [40,41] reported that santamarine and reynosin showed skin protective effects in both human dermal fibroblasts and keratinocytes. They suggested that santamarine and reynosin protected skin cells from changes caused by ultraviolet irradiation. The mechanism of action was inhibiting the activation of MAPK signaling to reduce the effect of the UV-mediated alteration of transcriptional activities. Both compounds were able to reduce the phosphorylation of p38, JNK, and ERK to some extent. Cho et al. [42] showed that both compounds inhibited tumor necrosis factor-α in murine macrophages. They suggested an action mechanism through MAPK signaling where they hypothesized that sesquiterpenoids might bind the sulfhydryl groups of kinases [43]. Similarly, other sesquiterpenoids were also found to be able to suppress MAPK activation. Abood et al. [44] reported that leucodine inhibited adipogenesis and suppressed the PPARγ pathway in 3T3-L1 preadipocytes by blocking mitotic clonal expansion via diminished p27 expression as a result of suppressed activation of ERK MAPK. Ye et al. [45] isolated sesquiterpenoids from *Atractylodes macrocephala*, which exerted anti-tumor properties by blocking the growth of B16 melanoma cells. They proposed an action mechanism that involves the inhibition of the Ras/ERK signaling pathway. These reports are all in accordance with the results of the current study, further supporting the suggestion that reynosin and santamarine were able to hinder adipogenesis by intervening with MAPK activation.

## 4. Materials and Methods

### 4.1. Materials and Reagents

The reagents used in cell culture, maintenance, and differentiation, including phosphate-buffered saline (PBS), were purchased from Gibco BRL (Thermo Fisher Scientific, Waltham, MA, USA) unless otherwise noted. 3-[4,5-dimethylthiazol-2-yl]-2,5-diphenyltetrazolium bromide (MTT), dimethyl sulfoxide (DMSO), and isopropanol were purchased from Sigma-Aldrich (Merck, St. Louis, MO, USA). The NE-PER nuclear protein extraction kit, the BCA protein assay kit for total protein quantification, and the primary antibody against JNK were from Thermo Fisher Scientific (Thermo Fisher Scientific, Waltham, MA, USA). Primary antibodies against PPARγ (#2443), CCAAT/enhancer-binding protein (C/EBP) α (#2295), p38 (#8690), phospho(p)-p38 (#4511), ERK (#4695), and p-ERK (#4370), were obtained from Cell Signaling Technology (Cell Signaling Technology, Danvers, MA, USA), while the sterol regulatory element-binding protein 1c (SREBP1c) antibody (ab3259) was from Abcam (Abcam Inc., Cambridge, UK). Other primary antibodies and horseradish peroxidase-conjugated secondary antibodies for Western blotting were purchased from Santa Cruz Biotechnology (SCBT, Santa Cruz, CA, USA). The remaining solutions, solvents, and chemicals were from Samchun Chemical (Samchun Chemical Co., Ltd., Seoul, Republic of Korea) unless otherwise noted.

### 4.2. Isolation of Compounds from A. scoparia

The samples of *A. scoparia* were collected from the coast of Donggeomri, Gilsangmyeon, Ganghwagun, Gyeonggido, Korea on 15 September 2018. The samples were dried in the shade and were extracted using methylene chloride (CH_2_Cl_2_) and methanol, respectively. After solvent removal, the resulting residue was sequentially fractionated into n-hexane, 85% aq. MeOH, n-BuOH, and H_2_O solvent fractions based on solvent polarity. According to the screening results, the 85% aq. MeOH fraction (2.52 g) was subjected to chromatography on C_18_ silica gel with stepwise gradient combinations of MeOH and water (ranging from 50% to 100% aqueous MeOH). This process yielded seven subfractions designated Fr. 1 to Fr. 7. Among these seven fractions, Fr.3 (0.07 g), which was obtained from the 70% aqueous methanol solvent, was further separated by reversed-phase HPLC (YMC ODS-A, 60% aqueous methanol, 2 mL/min) using a Dionex P580 HPLC system equipped with a Varian 350 RI detector (Varian Inc., Palo Alto, CA, USA). Compounds reynosin and santamarine were isolated in amounts of 12.2 mg and 15.0 mg, respectively. The identification of these compounds was established using NMR spectral data acquired with a standard pulse sequence program on a 600 MHz Agilent NMR system (Agilent Technologies, Santa Clara, CA, USA) with 600 MHz for ^1^H and 125 MHz for ^13^C nuclei. The chemical structures of the isolated compounds were identified by comparing NMR spectroscopic data below:

Reynosin: ^1^H-NMR (600 MHz, CD_3_OD)δ6.00 (^1^H, d, J = 3.1 Hz, H-13), 5.49 (^1^H, d, J = 3.0 Hz, H-13), 4.95 (^1^H, s, H-15), 4.79 (^1^H, s, H-15), 4.10 (^1^H, t, J = 10.9 Hz, H-6), 3.49 (^1^H, dd, J = 11.6, 4.5 Hz, H-1), 2.61 (^1^H, tdd, J = 11.2, 3.1, 3.0 Hz, H-7), 2.32 (^1^H, ddd, J = 13.5, 4.9, 1.8 Hz, H-3), 2.24 (^1^H, br d, J = 10.9 Hz, H-5), 2.15 (^1^H, td, J = 13.5, 5.1, H-3), 2.08 (^1^H, m, H-8), 2.05 (^1^H, m, H-9), 1.80 (^1^H, m, H-2), 1.60 (^1^H, m, H-8), 1.56 (^1^H, m, H-2), 1.38 (^1^H, td, J = 13.3, 3.8, H-9), 0.80 (3H, s, H-14); ^13^C NMR (125 MHz, CD_3_OD) δ 171.7 (C-12), 145.1 (C-4), 141.3 (C-11), 117.3 (C-13), 110.2 (C-15), 81.5 (C-6), 78.8 (C-1), 54.1 (C-5), 50.7 (C-7), 44.2 (C-10), 36.9 (C-9), 34.8 (C-3), 32.1 (C-2), 22.4 (C-8), and 12.0 (C-14).

Santamarine: ^1^H-NMR (600 MHz, CD_3_OD)δ6.00 (^1^H, d, J = 3.2 Hz, H-13), 5.49 (^1^H, d, J = 3.1 Hz, H-13), 5.36 (^1^H, m, H-3), 4.01 (^1^H, t, J = 11.1 Hz, H-6), 3.61 (^1^H, dd, J = 10.1, 6.7 Hz, H-1), 2.57 (^1^H, tdd, J= 11.2, 3.1, 3.0 Hz, H-7), 2.39 (^1^H, d, J = 11.1 Hz, H-5), 2.32 (^1^H, m, H-2), 2.09 (^1^H, m, H-8), 2.02 (^1^H, dt, J = 13.2, 3.4, H-9), 1.97 (^1^H, m, H-2), 1.66 (^1^H, m, H-8), 1.33 (^1^H, td, J = 13.2, 3.3 Hz, H-9), 1.81 (3H, s, H-15), 0.87 (3H, s, H-14); ^13^C NMR (125 MHz, CD_3_OD) δ 172.8 (C-12), 141.0 (C-11), 134.6 (C-4), 122.8 (C-3), 117.1 (C-13), 83.5 (C-6), 75.8 (C-1), 52.4 (C-5), 52.2 (C-7), 42.2 (C-10), 35.6 (C-9), 33.5 (C-2), 23.6 (C-15), 22.1 (C-8), and 11.4 (C-14).

### 4.3. Cell Culture and Maintenance

The 3T3-L1 preadipocytes used in the experiment were cultured in Dulbecco’s modified Eagle’s medium (Welgene, Gyeongsan-si, Republic of Korea), supplemented with 10% fetal bovine serum (FBS) and a 1% L-glutamine penicillin-streptomycin solution at 37 °C and under 5% CO_2_ environment conditions. Differentiation of preadipocytes into adipocytes was induced when the cells became confluent by replacing the culture medium with a differentiation medium, DMEM, containing 0.25 μM dexamethasone, 0.5 mM 3-isobutyl-1-methylxanthine, and 5 μg/mL insulin. Next, every 2 days, the existing medium was replaced with DMEM medium containing 10% FBS and 5 μg/mL insulin, and the degree of differentiation was observed for a total of 8 days.

### 4.4. Cytotoxicity Measurement

Cell viability was assessed using a modification of the method developed by Mosmann [46]. 3T3-L1 preadipocytes were plated at a concentration of 1 × 10^4^ cells per well in a 96-well plate and treated with various concentrations of reynosin and santamarine for 24 h. Subsequently, the cultured cells were rinsed with a fresh medium and exposed to a 1 mg/mL solution of MTT for a 4 h incubation period. After removing the MTT solution from the wells, the resulting formazan crystals were dissolved by adding 50 μL 100% dimethyl sulfoxide (DMSO). The absorbance of the wells was measured at 540 nm using a Multiskan GO microplate reader (Tecan, Grodig, Austria) to quantify the formazan formation as a means to evaluate cell viability.

### 4.5. Lipid Accumulation

Oil Red O staining was conducted to observe and quantify the intracellular lipid accumulation. The cultured and differentiated adipocytes on day 8 of differentiation were washed with phosphate-buffered saline (PBS) and fixed onto wells by adding 4% paraformaldehyde. They were then allowed to incubate at room temperature for 1 h. After removing the paraformaldehyde, the cells were stained with the Oil Red O solution and then washed with PBS. Once the washed cells were completely air-dried, the images of lipid droplets were taken, after which 100% isopropanol was added to the wells to extract the retained dye. The level of intracellular lipid accumulation was assessed by measuring the absorbance of the wells at 500 nm with a Multiskan GO microplate reader (Tecan, Grodig, Austria).

### 4.6. mRNA Expression

After culturing and differentiating 3T3-L1 preadipocytes in a 6-well plate, along with varying concentrations of samples the cells were washed with PBS, intracellular total RNA was extracted using an AccuPrep Universal RNA extraction kit (Bioneer, Daejeon, Republic of Korea) on day 8 of differentiation. Subsequently, cDNA was synthesized from the extracted RNA using the CellScript cDNA Master Mix kit (CellSafe, Yongin, Republic of Korea) following the manufacturer’s instructions. The synthesized cDNA was then amplified using a Luna Universal qPCR kit (New England Biolabs, Ipswich, MA, USA) according to the manual, and the products of amplifications were analyzed using a TP800 Thermal Cycler Dice™ Real Time System (Takara Bio, Ohtsu, Japan). Regarding the PCR cycling conditions, the process involved an initial denaturation step at 95 °C for 1 min, followed by denaturation at 95 °C for 15 s and extension at 60 °C for 30 s. These steps were repeated for a total of 40 cycles. The housekeeping gene β-actin was used for each sample, and the gene expression level was calculated using the delta-delta-Ct (∆∆Ct) method, employing the Ct values. The primer nucleotide sequences of the genes tested are provided in Table 1.

### 4.7. Protein Expression

After culturing 3T3-L1 preadipocytes in a 6-well plate, differentiation into adipocytes cells was induced to differentiate into adipocytes, as previously described, with varying concentrations of the samples. The differentiated cells on day 8 of differentiation were then washed with PBS, and total proteins were extracted from each well using RIPA buffer (Sigma-Aldrich, St. Louis, MO, USA). The protein concentration was quantified using a BCA protein assay kit (Thermo Fisher Scientific, Waltham, MA, USA). Subsequently, 20 μg of protein from each test group was separated through electrophoresis using sodium dodecyl-sulfate polyacrylamide gel electrophoresis (SDS-PAGE) and transferred onto a polyvinylidene fluoride membrane (Amersham Protran Biotech, Marlborough, MA, USA). Membrane blocking was carried out with the addition of 5% skim milk onto the membrane at room temperature for 1 h. Following membrane blocking, the membrane was washed and incubated with the primary antibodies for 24 h at 4 °C. Next, the horseradish peroxidase-conjugated secondary antibodies were applied. Protein expression was confirmed by imaging the membrane and using a Davinchi-Chemi Imager image analysis device (Davich-K, Seoul, Republic of Korea) to densitometrically quantify the bands in conjunction with the Supersignal West Femto Maximum Sensitivity Substrate chemiluminescence system (Thermo Fisher Scientific, Waltham, MA, USA) following the manufacturer’s instructions.

### 4.8. Statistical Analysis

To ascertain the significance of the results obtained from both the control group and each sample, a one-way analysis of variance (ANOVA) was conducted, followed by a comparative analysis using Duncan’s multiple range test at a significance level of *p* < 0.05. All experimental results were presented as the mean ± standard deviation (mean ± SD, *n* = 3). Statistical analysis was performed using the Statistical Analysis System v9.2 (SAS Institute Inc., Cary, NC, USA) software.

## 5. Conclusions

The current study reported that reynosin and santamarine isolated from *A. scoparia* inhibited the adipogenic differentiation of 3T3-L1 preadipocytes via suppressing MAPK activation, which was suggested to suppress the PPARγ-mediated activation of adipogenesis, resulting in diminished adipogenic characteristics. Overall, these results indicated that *A. scoparia* was a potential source of anti-adipogenic substances, reynosin and santamarine, and should be further investigated to elucidate their in vivo action mechanisms. Also, further studies using different cell lines, especially human-derived progenitor cells and in vivo models, would provide further insight into the anti-obesity potential of *A. scoparia* and the sesquiterpenoids reynosin and santamarine.

## Figures and Tables

**Table 1:** Sequences of primers used for reverse transcription semi-quantitative polymerase chain reaction (RT-qPCR).

Gene		Primer Sequence
PPARγ	Forward	5′-TTT-TCA-AGG-GTG-CCA-GTT-TC-3′
	Reverse	5′-AATCCT-TGG-CCC-TCT-GAG-AT-3′
SREBP1c	Forward	5′-TGT-TGG-CAT-CCT-GCT-ATC-TG-3′
	Reverse	5′-AGGGAA-AGC-TTT-GGG-GTC-TA-3′
C/EBPα	Forward	5′-TTA-CAA-CAG-GCC-AGG-TTT-CC-3′
	Reverse	5′-AAG-GAA-GGC-TGG-AAA-AGA-GC-3′
β-actin	Forward	5′-CCA-CAG-CTG-AGA-GGG-AAA-TC-3′
	Reverse	5′-AAG-GAA-GGC-TGG-AAA-AGA-GC-3′

## Data Availability

The data used in the current study are available from the corresponding author upon reasonable request.
